# Evaluation of a Digital, Self-Administered, Cognitive Test Battery in Older Adult Patients Undergoing Abdominal Surgery: Nonrandomized Feasibility Trial

**DOI:** 10.2196/71911

**Published:** 2025-11-07

**Authors:** Anahita Amirpour, Markus Saarijärvi, Jeanette Eckerblad, Gabriela Markovic, Anders Thorell, Ulrica Nilsson, Lina Bergman

**Affiliations:** 1 Department of Neurobiology, Care Sciences and Society, Karolinska Institutet, Alfred Nobels Alle 23, C4, Huddinge, 14152, Sweden; 2 Department of Cardiology, Danderyd University Hospital, Stockholm, Sweden; 3 Department of Neurobiology, Care Sciences and Society, Karolinska Institutet, Huddinge, Sweden; 4 Department of Rehabilitation Medicine, Department of Clinical Sciences, Danderyd University Hospital, Karolinska Institutet, Danderyd University Hospital, Stockholm, Sweden; 5 Department of Surgery and Anesthesiology, Department of Clinical Sciences, Ersta Hospital, Karolinska Institutet, Danderyd University Hospital, Stockholm, Sweden

**Keywords:** colorectal surgery, feasibility studies, neurocognitive disorders, neuropsychological tests, postoperative cognitive complications, user-centered design

## Abstract

**Background:**

Older adults undergoing surgeries face increased risks of postoperative neurocognitive disorders, which impair cognitive functions. Analog neurocognitive tests are commonly used, but digital tests offer faster, more accessible assessments.

**Objective:**

The primary aim of this study was to evaluate the feasibility of a digital cognitive test battery in older adults undergoing abdominal surgery. Feasibility included estimation of recruitment and retention rates, acceptability, perceived value, and usability of the test. The secondary aim was to explore outcome trajectories of cognition, depression, functional status, and quality of recovery.

**Methods:**

This nonrandomized feasibility study measured recruitment and retention rates using patient logs and expanded on these findings in semistructured interviews with nurses. Acceptability, perceived value, and usability were explored through interviews with patients and nurses, and the System Usability Scale (SUS). Cognitive functions were assessed with a digital cognitive test battery (Consortium to Establish a Registry for Alzheimer Disease [CERAD] word list learning test, Trail Making Test Parts A and B, Victoria Stroop Test, and Symbol Digit Pairing Test) and the Nursing Delirium Screening scale (NU-DESC), and depression with the Geriatric Depression Scale (GDS-15). Functional status was measured using the World Health Organization Disability Assessment Schedule (WHODAS), and postoperative recovery with the Swedish Quality of Recovery questionnaire (SwQoR-24). Quantitative data were analyzed using descriptive statistics and nonparametric tests and qualitative data with content analysis.

**Results:**

The test battery was feasible, acceptable, and demonstrated excellent usability. The mean SUS score was 87 (SD 17.9; 95% CI 78.9‐95.2), and all predefined progression criteria were met. Recruitment spanned over 1.5 years, during which 24 patients were included (mean age of 77, SD 6.5 years; range: 63‐90 years; n=13, 54% women). Most patients underwent laparoscopic colorectal cancer surgery. Three patients developed postoperative delirium for 1 day only. No patient developed delayed neurocognitive recovery or mild/major neurocognitive disorder at the postoperative follow-up. Qualitative data showed that both nurses and patients regarded the digital cognitive test battery as important for assessing cognitive function and found it easy to use and understand. Nurses reported that recruitment was challenging, partly because not all patients attended a preoperative in-person consultation before surgery.

**Conclusions:**

The digital, self-administered cognitive test battery was found to be feasible, acceptable, and usable in older adults undergoing abdominal surgery. However, recruitment challenges and a small, homogeneous sample limit generalizability and warrant careful consideration in a larger-scale study.

## Introduction

Undergoing surgery as an older adult comes with its own set of challenges and risks. Older adults often experience a higher symptom burden compared to younger patients, and the aging process affects various biological and functional systems [1], including the brain and cognitive functions. Postoperative complications are a significant concern for older adults, with cognitive complications being particularly common. These include postoperative delirium (POD), delayed neurocognitive recovery (dNCR), and postoperative neurocognitive disorder (p-NCD) [2]. POD can occur within the first week after surgery, while dNCR may develop within 30 days. The effects of p-NCD, whether mild or major, can persist up to a year. These conditions can impact cognitive functions, leading to impairments in attention, memory, and executive functions [3], and manifest themselves as forgetfulness, difficulty initiating tasks, and challenges in executing daily activities [4].

Various methods exist for assessing cognitive performance following surgery, with analog tests being the most used [5]. Recommended tests typically focus on episodic memory, executive functions, and attention [6]. Digital tests offer faster screening and analysis of cognitive functions, reduction of administrative bias, and enhanced accessibility as they can be performed outside of clinical settings [7]. Despite its advantages over analog tests, digital tests remain underutilized in perioperative care.

One such digital test, Mindmore, provides self-administered cognitive test batteries grounded in established psychometric tests, including normative data adjusted for sex, age, and education [8]. The digital cognitive test batteries are validated, Conformité Européenne–certified, with demonstrated high usability among older adult volunteers [7] and are developed by licensed psychologists at a private company [7,8]. Research from other settings indicates that digital and remote assessments are generally feasible and acceptable among older adults [9-11], including smartphone-based monitoring [9], tablet tools for postoperative mobilization [10], and telephone-based cognitive testing [11]. However, no study has yet examined the feasibility of a digital, cognitive test with older adults undergoing surgery.

Thus, the primary aim of this study was to evaluate the feasibility of using a digital cognitive test battery in older adults undergoing abdominal surgery. Feasibility included estimation of recruitment and retention rates, acceptability, perceived value, and usability of the test. The secondary aim was to explore outcome trajectories of cognition, depression, functional status, and quality of recovery.

## Methods

### Overview

We followed the medical research council’s framework for evaluating complex interventions [12] and prospectively registered our study at clinicaltrials.gov (NCT05564195) and published our study protocol [13]. We report in accordance with reporting guidelines for nonrandomized feasibility studies [14] and relevant items from the CONSORT (Consolidated Standards of Reporting Trials) 2010 statement extension for pilot and feasibility trials [15].

### Study Design and Setting

We conducted a nonrandomized single-group feasibility study with the purpose of assessing key components for a future, larger cohort study [14]. The study was carried out from January 2023 to November 2024 in Stockholm County, Sweden. The hospital where the patients were recruited specializes in abdominal surgery and handles approximately 4700 outpatient visits annually.

### Study Participants and Procedure

Registered nurses at the surgery clinic and research unit screened and identified potential participants who met the inclusion criteria at the preoperative visit, provided study information, and obtained written informed consent. Inclusion criteria were patients ≥60 years undergoing planned surgery lasting a minimum of 60 minutes. Exclusion criteria included Mini-Mental State Examination (MMSE) score <23, current psychiatric disease (eg, bipolar disorder), central nervous system disease (eg, stroke), uncorrected hearing or visual impairments, motor impairment in the dominant hand, defective color vision, and lack of proficiency in the Swedish language. A target sample size of 50 participants was planned, based on a recommended minimum of 30 participants for pilot studies to provide initial estimates of outcome variability and feasibility for future trials [16]. A Swedish version of the MMSE was administered at the beginning of the study before obtaining written permission from Psychological Assessment Resources (PAR). This administrative oversight was later corrected, and the required authorization has since been secured. The MMSE is a copyrighted instrument and may not be reproduced or used in any form or language without prior written permission from PAR.

### Data Collection

Quantitative data investigated recruitment and retention process, usability, cognitive performance, including clinician and patient-reported outcome measures. We collected data at baseline (T0) and postoperatively including follow-ups at the hospital 1‐3 days (T1), 3‐5 weeks (T2), and at 6 months (T3) at the patient’s home or at the premises of Karolinska Institutet. All the outcome measurements included are presented in Table 1. As some instruments lacked validation in older adults in a perioperative context, their use in this study was exploratory.

**Table 1.** Included clinician-reported and patient-reported outcome measurements.

Questionnaire	Outcome	Properties	Timepoint	Validation in Swedish context
Clinical Frailty Scale (CFS) [17]	Frailty	9-item, ranging from 1-very fit to 9-terminally ill. Very fit means people who are robust, active, energetic, and motivated.	T0^a^	Validated in patients 65 years and older [18].
Nursing Delirium Screening Scale (NU-DESC) [19]	Delirium	5-item, observational scale where each symptom is rated 0‐2. A score of ≥2=delirium.	Each postoperative day at the hospital	Validated in older adult patients undergoing cardiac surgery [20].
Swedish Quality of Recovery (SwQoR-24) [21]	Postoperative quality of recovery	24-item, including rating pain, anxiety, and sleep difficulties. The items are rated on an 11-point Likert scale ranging from 0=none of the time to 10=all the time. The global score ranges from 0‐240 where scores ≥32 indicate poor recovery.	T0 T2^b^ T3^c^	Validated in patients undergoing surgery [22].
Geriatric Depression Scale (GDS-15) [23]	Depression	15-item consists of binary (yes/no) questions on depressive symptoms. Scores at ≥5 indicate depression.	T0 T2 T3	Validated in older adult patients [24], .
System Usability Scale (SUS) [25]	Usability	10-item, on a five-point Likert scale. The score ranges from 0‐100, where a higher score indicates high usability.	T2	Not validated. We used a Swedish translated version and slightly modified the statements to “tablet” instead of “system” to enhance participant comprehension.
World Health Organization Disability Assessment Schedule (WHODAS 2.0, 12-item)	Function	12-item, ranging from 1=none to 5=extreme, reflecting the degree of difficulty experienced in various functional areas. The score ranges from 12 to 60, with a score of 12 indicating “none” for all items.	T0 T2 T3	Validated in patients with psychotic disorders [26].

a T0: baseline, preoperatively.

b T2: 3-5 weeks postoperatively.

c T3: 6 months postoperatively.

Qualitative data explored the acceptability, usability, and perceived value of the digital cognitive test battery by collecting data from 2 sources:

- Face-to-face or video interviews via Zoom (Zoom Video Communications) with 17 patients at the patient’s home. Patients chose their preferred mode of interview. Data collection took place after T2, except for one patient, whose interview was conducted at 5 months after surgery.
- Video interviews via Microsoft Teams (Microsoft Corporation) or Zoom with 5 nurses at the research unit and surgery clinic, recruiting patients and delivering the digital cognitive test battery. Data collection took place after T2.

The first author (AA) conducted the interviews with the patients, and the last author (LB) conducted the interviews with the nurses. We developed and followed semistructured interview guides (Multimedia Appendix 1) for all interviews, which contained open-ended questions and suggestions for additional probing questions. The interview guide for the nurses was based on Sekhon et al’s [27] framework for acceptability, which includes 7 constructs: affective attitudes, burden, ethical aspects, intervention coherence, opportunity costs, perceived effectiveness, and self-efficacy. After the initial interviews, the guide was discussed and evaluated within the research group. Each interview was audio-recorded and transcribed verbatim by a transcription company.

### Cognitive Test Battery

Mindmore offers different cognitive test batteries depending on the patient group and procedure [7]. The tests included in this study are listed in Table 2. Each test begins with an introduction, followed by a practice trial with feedback and then the actual test trial. The 4 tests take approximately 15 minutes to complete and are conducted on a 10.1-inch Windows touchscreen study-specific tablet. The capacitive touchscreen records detailed information such as timing, pauses, and finger lifts from the screen. Further, speech recognition is used for screening verbal memory. The patients underwent the cognitive test battery at T0, T1, T2, and T3.

**Table 2.** Included cognitive test battery.

Cognitive test	Cognitive domains	Properties
Consortium to Establish a Registry for Alzheimer Disease (CERAD) word list learning test	Verbal episodic memory, delayed recall	10-word verbal learning test conducted over three trials, followed by recall around 7 minutes later. For the word list learning trial, the score ranges from 0‐30, where a higher score indicates a higher function. For delayed recall, the maximum score is 10.
Trail Making Test (TMT A and B)	Attention, processing speed, visuospatial function, executive functions	25 circles in each segment, requiring the participants to connect them sequentially. In Part B, the participants must connect 13 numbers and 12 letters alternately. A higher number of connections and shorter time indicate higher performance.
Victoria Stroop Test	Executive functions, selective attention, cognitive flexibility, inhibition response	24 words depicting different colors, printed using contrasting ink colors. The participant is directed to quickly and thoroughly identify the color of the ink rather than the word itself. Test score is calculated as an index: meaning number of correct answers and average time, and for interference.
Symbol Digits Processing Test (SDPT)	Attention, processing speed	9 randomized symbols to reduce practice effects. The participant matches the symbols to digits on a 3x3grid within 90 seconds, with the final score based on correct matches.

### Outcomes

An overview of the feasibility outcomes and progression criteria [28] is presented in Table 3. Our progression criteria (eg, warranting a recommendation to proceed to a larger trial) were based on our previous research with patients undergoing total hip arthroplasty [29] and previous studies including patients undergoing colorectal surgery [30,31]; consequently, we set the following progression criteria: recruitment ≥20% of available patients, dropouts ≤30%, and usability ≥75 on the System Usability Scale.

**Table 3.** Feasibility outcome measures.

Outcome	Description	Progression criteria	Data source
Study design, recruitment, and retention	Estimates, reasons for nonparticipation and dropouts	≥20% of available patients; dropouts <30%	Patient screening log; semistructured interviews
Acceptability and perceived value of the digital cognitive test battery, by patients and nurses	Experiences of using the digital cognitive test battery	—^a^	Semistructured interviews
Usability of the digital cognitive test battery, by patients and nurses	System Usability Scale; experiences of using the digital cognitive test battery	System Usability Scale Score ≥75	System Usability Scale; semistructured interviews

a Not applicable.

### Statistical Analysis

Descriptive statistics included numbers and percentages for categorical variables, means, median, SD, for continuous variables, and 95% CIs where appropriate. Analysis of normal distribution was performed with Q-Q plots, histograms, and the Shapiro-Wilk test. We applied the nonparametric Friedman test on repeatedly measured data. We defined cutoffs for dNCR/p-NCD following the recommendations by Borchers et al [6]: mild decline at 1 to 2 SD and major decline >2 SD in at least 2 test parameters at postoperative time points 3‐5 weeks or 6 months. We considered a 2-sided *P* value of <.05 as statistically significant. All statistical analyses were performed in IBM SPSS version 28 (IBM Corp).

### Qualitative Analysis

Three authors with experience in qualitative methods (AA, MS, and LB) analyzed the qualitative data through an iterative process. We conducted a deductive content analysis as described by Kyngäs and Kaakinen [32] by applying the theoretical framework of acceptability [27] on our data. Initially, we created an analysis matrix based on the 7 constructs of acceptability and added a dimension for study design, feasibility, and recruitment. Authors AA, MS, and LB thoroughly read each transcribed interview multiple times to familiarize themselves with the data and assess the depth of information. LB organized the data using NVivo (version 15, QSR International Pty Ltd), and coded quotes related to our research questions using the analysis matrix. AA validated the codes independently in NVivo. Subsequent discussions with MS ensured coding decisions and researcher triangulation. MS created categories inductively according to the constructs in our matrix, with AA and LB reviewing and refining them collaboratively. AA translated the final meaning units from Swedish to English, and LB selected illustrative quotes for inclusion. Discussion of initial categories and reflections with the co-authors of the manuscript led to the re-organization of our meaning units and categories within the matrix. All authors’ diverse clinical and research backgrounds, including anesthesia, surgery, postoperative care, intensive care, and neuropsychology, provided complementary perspectives during analysis. The content analysis is presented in Table 4.

**Table 4.** The process of content analysis.

Text quote examples	Categories	Domains
”[…] it’s actually okay to contact patients, […] to come for an extra visit, just to participate in a study, because the patients who’ve been involved have, at least most of them, thought it was very fun.” (RN4)	Barriers and facilitating factors in selection, recruitment, and retention processes	Study design, recruitment, and retention
”I thought it was interesting... […], this study you’re doing, […] to see how it affects you, because […] I hadn’t thought that a physical procedure would have such a big psychological impact as I understood it would have. […] I had never reflected on it in that way, but I was more... thought that a physical procedure is a physical procedure and then you move on […]” (P23)	Participants’ feelings and attitudes toward the intervention	Affective attitudes
“I thought it was easy... actually easy to understand. There weren’t any practical difficulties at all with it, I thought. However, some parts might have been difficult to carry out at different times. But overall, I thought it was very simple, clear, and good.” (P20) “But it’s a […] patient group that has cancer. […] they are a group that you must be a little more careful with, especially if they’ve just received a diagnosis, and... Some are, like... they are right there and want to […] whereas many are like, ‘No, I can’t take anything more from anyone, because I’ve just gotten this news. I have so much.’ So, […] you have to be a little more, not too... well, careful with, maybe, a little... […] A little more sensitive, I think […] there are some who’ve felt they haven’t had the energy, because they’ve had it so tough with so many other things, you know, around their surgery, preparations, and their cancer, and everything.” (RN1)	No sense of burden Illness- and symptom related factors	Burden

### Ethical Considerations

We obtained an ethical permit (2022-03593-01) from the Swedish Ethical Review Authority before initiating the study. All procedures complied with the World Medical Association’s Declaration of Helsinki. Written informed consent was obtained from all participants before inclusion. Data were pseudonymized to ensure confidentiality. Participation was voluntary, without compensation, and participants could withdraw at any time.

## Results

### Participant Characteristics

Twenty-four patients were included in the study: 13 women and 11 men. The mean age was 77 (SD 6.5) years. Half of the patients (12/24, 50%) were classified as “very fit,” and none were classified as frail according to the Clinical Frailty Scale (CFS). Most of the patients had obtained a university degree and had an advanced occupational background: two patients were still working at the time of data collection. Most patients (20/24, 83%) underwent laparoscopic colorectal surgery for cancer under general anesthesia combined with spinal anesthesia. Eight patients developed postoperative complications, such as wound infections. Two of these patients required resurgery, and one of them also needed intensive care. At T2, 3 patients were treated with or had recently completed chemotherapy, one patient had developed an autoimmune disease, and 2 had a recent infection. Patient characteristics are presented in Table 5. The 5 registered nurses interviewed were between 35 years and 60 years old, with 12-29 years of experience in their profession.

**Table 5.** Patient characteristics.

Characteristic	Total sample (N=24)
Sex, n (%)	
Female	13 (54)
Male	11 (46)
Age, years	
Mean (SD)	77 (6.5)
Min-max	63‐90
Level of education, n (%)	
Elementary school	4 (17)
Upper secondary school or similar	3 (12)
Post-secondary education	4 (17)
University education	13 (54)
Occupation ISCO-08^a^, n (%)	
Managers	8 (33)
Professionals	11 (46)
Technicians and associate professionals	3 (12)
Service and sales workers	2 (8)
Clinical Frailty Scale, mean (SD)	
Very fit	12 (50)
Well	7 (29)
Managing well	5 (21)
Mini-Mental State Examination, mean (SD)	29 (1)
American Society of Anesthesiologists classification^b^, n (%)	
I	4 (17)
II	11 (46)
III	8 (33)
Comorbidities before surgery, n (%)	
Cardiac disease	7 (29)
Vascular disease	2 (8)
Lung disease	4 (17)
Kidney disease	2 (8)
Diabetes	1 (4)
History of cancer	3 (13)
Autoimmune disease	2 (8)
Type of surgery^b^, n (%)	
Laparoscopic colorectal	20 (87)
Laparoscopic paraesophageal hernia	3 (13)
Type of anesthesia^b^, n (%)	
General anesthesia	3 (13)
General anesthesia and epidural	3 (13)
General anesthesia and spinal	17 (71)
Duration of surgery^b^	
Minutes, mean (SD)	190 (77)
Duration of anesthesia^b^	
Minutes, mean (SD)	258 (86)
Intraoperative bleeding^b^	
Milliliter, mean (SD)	28 (33)
Length of hospital stay^b^, mean (SD)	5 (4)
Postoperative complications^b^, n (%)	8 (33)
Resurgery^b^, n (%)	2 (8)

a ISCO-08 = International Standard Classification of Occupations.

b One missing.

### Feasibility

The set progression criteria were met and the digital cognitive test battery was deemed feasible; System Usability Scale Score ≥75, recruiting ≥20% of available patients and dropouts <30%.

### Recruitment and Retention

**Figure 1.**
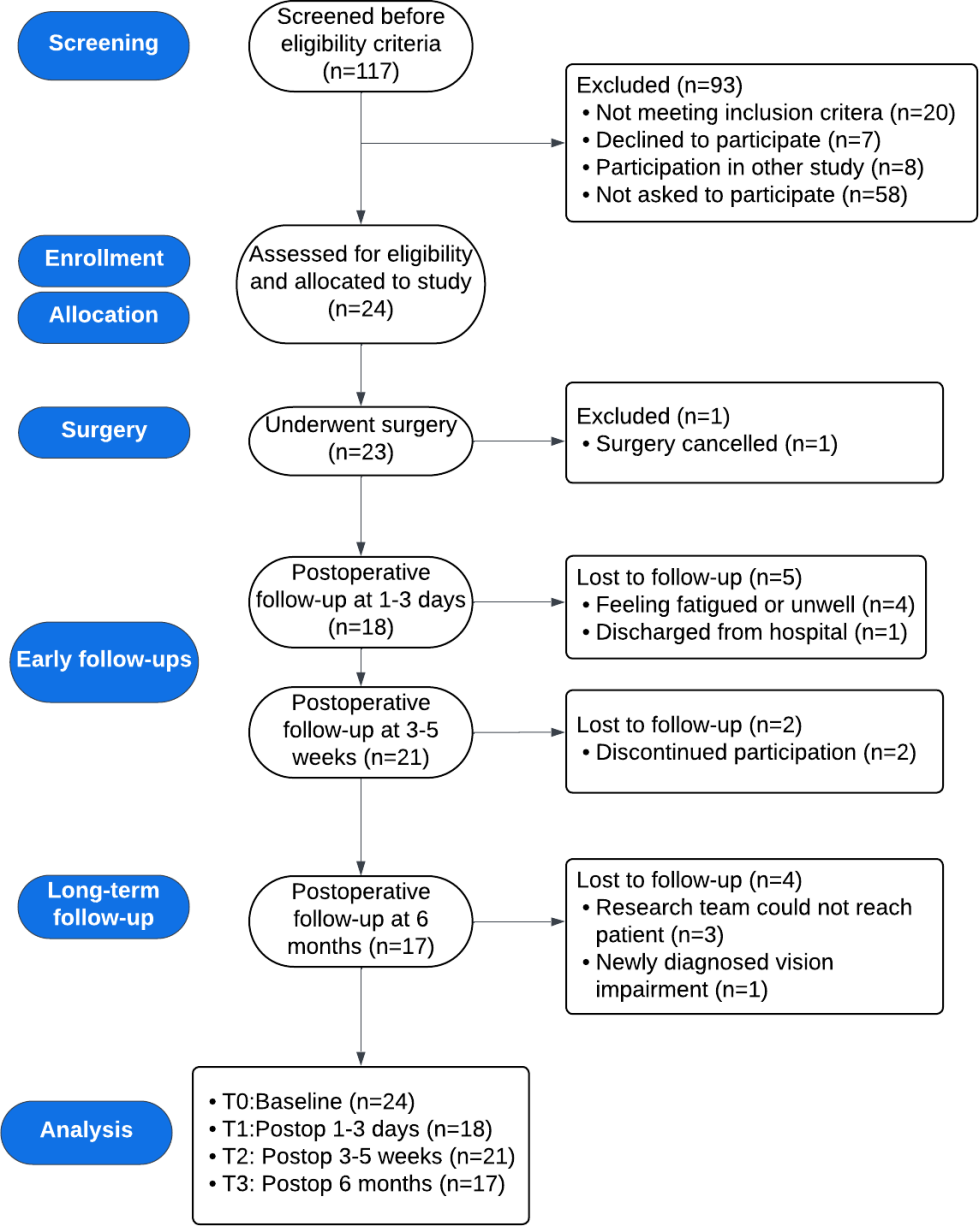
Study flowchart of participants from screening to study completion. Follow-up assessments were conducted postoperatively at 1‐3 days, 3‐5 weeks, and 6 months. Numbers of participants excluded, lost to follow-up, and analyzed at each time point are shown. Postop: postoperative follow-up.

Recruitment took place over a period of 1.5 years, from January 2023 to June 2024. Data collection concluded in November 2024. Out of 117 patients screened for eligibility (Figure 1), 20.5% (n=24) were included in the study, dropout rates were 12% at T2 and 27% at T3.

### Acceptability, Perceived Value, and Usability

Twenty patients completed the SUS at T2, the mean score was 87 (SD 17.9; 95% CI 78.9‐95.2), indicating excellent usability [33]. The qualitative results are presented below.

#### Study Design, Recruitment, and Retention

Several key factors influencing recruitment success were described by the nurses: the research team being available, and engaging with the clinical staff about the study, good collaboration between the research unit and the nurses at the surgery clinic, and that the study did not require any extra visits to the hospital. The nurses found it easy to approach patients for participation since the study was not information heavy. They emphasized that clear written and verbal communication from the research team was beneficial and improved the collaboration between the researchers and the clinic.

The nurses at the surgery clinic were the first to meet the patients and therefore played an important role in the recruitment process. Positive attitudes among the nurses also contributed to a favorable recruitment environment. However, several barriers were also identified. These included time constraints, as nurses sometimes had no opportunity to approach patients during the in-person preoperative consultation, as other commitments took priority. Some patients did not attend an in-person preoperative consultation at all, meaning they were not approached for participation. According to the nurses, some participants, particularly older adults, may struggle with using a tablet, which in some cases led nurses to refrain from inviting them to participate. The nurses also emphasized that the patients constituted a vulnerable group due to their cancer diagnoses. At times, the nurses felt it was inappropriate to invite these patients to participate in the research study for that reason.

*Yes, absolutely [about hesitating to ask the patient to become a study participant]. […] if you feel the patient is struggling to process all the information you’ve already given about the care and what will happen, and then you feel like, “No, this is too much. This patient isn’t receptive to this.” Or if they’re having a crisis reaction or if you assess that, “No, this isn’t appropriate to ask outright.”*

[RN5]

Nurses also noted that clinical staff at the postoperative wards were unfamiliar with the delirium assessment, requiring repeated reminders and instructions from the research nurses. In addition, the nurses felt that the research team’s expectations for recruitment numbers (50 patients) were unrealistic. Recruitment was further challenged by competition from another study targeting the same patient population, which meant some patients were approached only for the competing study. Suggestions from the nurses for improving the recruitment included clearer inclusion and exclusion criteria, flexibility in timing of inclusion, and better patient preparation, such as info available in the waiting room. The hospital also relocated to another building while the study was ongoing, which also affected recruitment.

#### Affective Attitudes

Patients expressed largely positive and curious feelings toward the digital test battery, describing it as interesting, enjoyable, and meaningful. It was perceived as easy to use and unproblematic. The patients also appreciated having the chance to do something else for a while. They also valued the opportunity to reflect on cognitive and psychological aspects of surgery. Many patients had not considered that surgery could impact their brains, as their attention was mainly focused on abdominal symptoms.

*I thought it was interesting… […], this study you’re doing, […] to see how it affects you, because […] I hadn’t thought that a physical procedure would have such a big psychological impact as I understood it would have. […] I had never reflected on it in that way, but I was more… thought that a physical procedure is a physical procedure and then you move on […]*

[P23]

At the same time, some patients described an anticipatory anxiety about the cognitive tests, particularly when they were already mentally burdened by their diagnosis or treatment. The nurses perceived that participants enjoyed taking the tests, as it allowed them to focus on focus on something different and get in the zone. They also noted that the patients valued the extra attention they received as they were also study participants, which seemed to increase motivation.

*[It was] a very pleasant meeting with the patient […] and I think they’ve felt that too, that it’s been a little […] extra thing, […]. Both those who have been seriously ill and those who have [been feeling fine]. And it’s been a bit different. […] they’ve still thought it was fun, […] and they’ve been happy to do it [the cognitive test].*

[RN2]

However, some nurses observed that certain participants found specific tests challenging or stressful. Despite this, the nurses themselves were satisfied with the digital cognitive test battery and appreciated the interactions with patients, noting only minor technical difficulties in one subtest. They also felt responsible for explaining to patients why the tests were intentionally designed to be challenging.

#### Burden

Participants’ experiences of burden varied depending on individual circumstances and the timing of assessments. Several participants described no burden at all; the tests required minimal effort. They perceived the cognitive tests as easy to complete and as one of many and comparable to other routine activities within the care process. Some patients even approached the tests competitively, motivated to perform well.

*I thought it was easy… actually easy to understand. There weren’t any practical difficulties at all with it, I thought. However, some parts might have been difficult to carry out at different times. But overall, I thought it was very simple, clear, and good.*

[P20]

For others, illness- and symptom-related factors influenced participation. Medical complications, fatigue from surgery or treatment preparation, and the overall cognitive strain of the perioperative process led participants to decline participation. Completing the tests shortly after surgery was described as cognitively demanding and exhausting. Nurses emphasized that patients with cancer diagnoses required extra attentiveness and support. They felt these patients often had a great deal to process regarding their diagnosis and treatments, and therefore needed to be approached with more care. The nurses also described that some patients experienced difficulties sitting upright after abdominal surgery due to their wound.

*Some patients have […] had a positive expectation and thought, “This sounds really exciting, now I’ll try this,” while some have been more, like, anxiety-filled, “Oh no, yeah, should I participate…? This will be tough…” That is, some… some maybe aren’t feeling great to begin with, and it feels like they… “Well, now am I also going to find out that I’m not quite sharp in the head anymore, now that my body has given up? Are we now going to discover that I can’t manage this test?” […] I remember having patients, for example, who’ve had previous burnout […] and they’ve felt like, “No, I don’t want to do this, because I don’t want to add any stress about how my brain functions.”*

[RN3]

Intervention-specific factors also played a role. The nurses suggested that a simpler, less extensive cognitive test battery would be more feasible in the perioperative setting. Nonetheless, they observed that participation did not represent a major sacrifice for most patients.

#### Ethical Aspects

The participants viewed the study as meaningful and expressed a strong desire to contribute, even when faced with numerous other commitments around the time of surgery. Those who chose to participate were motivated by the opportunity to support research and potentially benefit future patients. According to the nurses, most patients who were invited agreed to participate.

*[…] But when you notice that someone… has impaired cognition, maybe not just from the surgery, but actually for another reason and would need a dementia assessment. What do you do then? It becomes another… [about the responsibility to provide feedback as a clinician].*

[RN4]

At the same time, some nurses highlighted an ethical dilemma concerning the handling of potentially negative cognitive test results. Because they did not routinely work with cognitive assessments, they felt uncertain about how to respond if the cognitive tests would be implemented at their clinic.

#### Intervention Coherence

Several factors facilitated test compliance. Participants described that the instructions on the digital cognitive test battery were very clear and easy to understand, even if some were not used to using a tablet. The patients described that they thought it could be implemented in the perioperative process as they found it interesting.

*I’ve never worked with a tablet like that. I use a regular computer. I’m not so good with my phone either, but with browsing around, it’s the computer. But it worked with that tablet. […] when I was actually sitting there, I went into that bubble.*

[P15]

The nurses emphasized the importance of explaining that the tests were intended to be challenging but not competitive, in order to prevent participants from feeling inadequate. The timing of assessments also influenced participants’ engagement: tests administered 1‐3 days postoperatively were difficult to complete due to fatigue, whereas later follow-ups were easier as participants had more energy. There were also other barriers and risks to the execution and implementation of the cognitive test battery:

*[…] it requires that all the staff who handle them, you know, get proper training on how it works. I don’t think you can just throw it in and say, “Here, give this to a patient who’s going to do this,” you have to[…] do it carefully, just like with any research study, you need to implement it properly.*

[RN1]

Since the tests were self-administered, the nurses sometimes felt less involved. They noted; however, that some patients required additional support during the test session and appreciated the nurse’s presence. Nurses highlighted that comprehensive staff training would be essential for successful implementation if the tests were to be used more widely in the clinic.

#### Opportunity Costs

The patients viewed participation in the study as a positive investment in their cognitive health. They found it interesting to learn about their cognitive functions and considered the sessions a worthwhile trade-off, often describing them as “just another thing on the to-do list”

*[…] it’s also interesting to know how their cognitive abilities are after the surgery, so it’s a balancing act there, […] But I think… otherwise, I think it has been good.*

[RN1]

One patient described a situation where a nurse appeared stressed while administering the test. The nurses emphasized that staff need sufficient time both to administer the tests and to ensure they are completed in a proper manner.

#### Perceived Effectiveness

Participants appreciated both the cognitive and clinical value of the digital cognitive test battery. The nurses noted that, if resources allowed, the tests could potentially be integrated into routine care. From a cognitive perspective, patients found the tests stimulating, though some tests were highly challenging, which made them aware of their own cognitive limitations.

*And I didn’t quite realize, you know, what shortcomings or… what deficiencies I have. […] that was a bit exciting, and a bit revealing that in some respects, I’m as inattentive as I am. So, I thought that was… it was an interesting experience.*

[P23]

Some patients described that repeated completion of the tests allowed them to learn and improve over time. They also described feeling that their “brains were activated,” which inspired them to engage in continued cognitive training outside the study. A few patients noted that the tests gave them a sense of what memory or dementia assessments would be like.

#### Self-Efficacy

The nurses described that some patients needed additional support during the test sessions to move forward with each test, as some instructions were perceived as unclear at times.

*[…] most of them, I think, need a test leader with them. It’s a bit age-related too. The ones who are a bit younger and more familiar with technology. Because some can already say […] “Well, I can’t… I’m so bad with all this technology. I won’t be able to manage this.” But then when you explain, “This is it. You just have to follow the instructions […]” For many, especially if they are older, can be a bit scared of this technology, which isn’t about filling out papers. […] they probably would manage it quite well on their own, but they felt a sense of security knowing there was a person beside them.*

[RN1]

However, most patients managed the test session fully independently. The patients also described that they felt confident in their abilities to complete the tests, without extra assistance.

### Cognitive Trajectories

Three patients fulfilled the criteria of POD for only one day while in the surgical ward: 2 on the first postoperative day and one on the third postoperative day. No further cases of POD were observed during the subsequent postoperative days in the hospital. There were statistically significant changes in cognitive test results between the preoperative and postoperative follow-ups in the attention and tempo domain and the executive functions domain. The patients’ *z* scores are presented in Multimedia Appendix 2. No patients met the criteria for dNCR or p-NCD at the postoperative follow-ups. Cognitive raw scores from the different data points are presented in Multimedia Appendix 3.

### Trajectories of Depression, Functional Status, and Quality of Recovery

Preoperatively, one patient fulfilled cut-off criteria for depression, 4 patients at T2, and one at T3. However, on a group level, the GDS scoring was low overall. Functional status scores with World Health Organization Disability Assessment Schedule (WHODAS) 12 2.0 were highest at T2. The patients’ highest scoring symptoms on the Swedish Quality of Recovery questionnaire (SwQoR) were in the cognitive and emotional domain eg, worry, concentration, and well-being. There were no statistically significant changes between preoperative and postoperative measurements of depression, functional capacity, and quality of recovery (Multimedia Appendix 2).

## Discussion

### Principal Findings

This was a nonrandomized feasibility study aimed at assessing a digital, self-administered cognitive test battery in older adults undergoing abdominal surgery. Recruitment and retention proved challenging, with rates falling below expectations. The 1.5-year recruitment period turned out to be unfeasible, and a single-center approach is not viable for a larger-scale trial. One contributing factor to the shortfall in sample size was that 58 eligible patients were not approached for participation, in part because some did not attend a preoperative in-person consultation before their scheduled surgery. Similar recruitment challenges have been reported in feasibility studies involving patients with cancer diagnoses [10,34,35].

For a future trial, a multicenter recruitment model using both clinical and research staff may be necessary [36]. Consent to participate can be performed remotely via an invitation letter or email to the patient before the preoperative consultation [36], and eligibility screening could be performed remotely using validated tools, such as the telephone-administered Montreal Cognitive Assessment [37,38] instead of in-person MMSE. Online advertisement such as Google Search Engine may further expand reach [39].

Other contributing factors behind low recruitment rates were staff-related: nurses refrained from inviting some patients to participate. Their decisions were influenced by their perceptions of patient receptiveness, emotional considerations due to their cancer diagnosis which aligns with previous research in patients with lung cancer diagnoses [40], and patients’ familiarity with digital devices. Additional barriers included logistical disruptions, such as hospital relocation.

The participants highlighted barriers and facilitators to implementing cognitive assessments in the perioperative setting. Some nurses expressed uncertainty about how to respond when a patient scored low on the cognitive tests, emphasizing the need for structured guidance and staff training. This aligns with findings from a study on cognitive assessments in primary care [41]. Ethical and implementation challenges can be addressed through staff education and decision-support interventions; previous evidence suggests these approaches have shown mixed success in improving management of cognitive disorders [42]. Moreover, factual knowledge alone may not be sufficient; addressing clinicians’s attitudes and implicit biases has also been shown to influence the management of cognitive disorders [43].

In terms of acceptability and usability, the digital cognitive test battery was considered easy to use and comprehend, with a high perceived value from both patients and nurses. Patients rated the digital cognitive test battery as having excellent usability, which aligns with previous findings [7]. Although cognitive screening is not performed routinely in older adults undergoing surgery, it provides actionable information to identify at-risk patients [44]. Cognitive test results indicating patients are at risk can guide preventative strategies, including implementation of the Hospital Elder Life Program for postoperative delirium [45], referral to geriatric specialists, or integration of a multidisciplinary perioperative care team [44].

No patient developed dNCR or p-NCD following surgery. The subtest assessing attention and processing speed improved over time; however, this finding must be interpreted with caution. Given the repeated exposure to the same tests, practice effects may contribute to observed improvement [46]. The small sample size and lack of a control group in our study constrain interpretation. Future trials should consider alternate versions of similar tests or control groups. Several patients had a high educational and occupational background, which reflects a cognitively robust sample. Patients with low cognitive reserves are at higher risk of developing postoperative cognitive disorders compared to those with high cognitive reserves [47,48].

This study had some strengths. To the best of our knowledge, this was the first time a digital, self-administered cognitive test battery was tested for feasibility in this clinical context. By deciding a priori progression criteria, we reduced the risk of designing an ill-informed larger trial in the future [28].

We acknowledge the limitations in this study. The cohort was relatively homogeneous and cognitively robust. This homogeneity limits the external validity, as feasibility in this group may not reflect outcomes in frailer or more diverse perioperative populations, particularly those with lower cognitive reserves. Moreover, the high usability scores should be interpreted cautiously, as they may not translate to patients with limited digital literacy. Additional limitations include the absence of a control group, which restricts interpretation of cognitive outcomes, as improvements in attention may partly reflect practice effects rather than true changes. Moreover, some of the clinician- and patient-reported outcome measurements used in this study have not been validated in this population and setting. Recruitment difficulties and the modest sample size posed additional limitations, and we acknowledge that recruitment was, in many cases, not feasible. We have a few inconsistencies with the published study protocol [13] before starting our study, as we extended the data collection and decided to include nurses to assess the acceptability and usability of the digital cognitive test battery.

### Conclusions

The digital, self-administered cognitive test battery was found to be feasible, acceptable, and usable in older adults undergoing abdominal surgery. However, recruitment challenges and the homogeneous sample limit the generalizability of these findings. Implementation in clinical practice would require structured staff training and ethical considerations. Future trials should include larger, more diverse samples, consider control groups or alternate test versions to account for practice effects in the cognitive tests.

## Supplementary material

10.2196/71911 Multimedia Appendix 1 Semistructured interview guides for patients and nurses.

10.2196/71911 Multimedia Appendix 2 Patients’ *z* scores and depression, functional, and quality of recovery scores.

10.2196/71911 Multimedia Appendix 3 Cognitive raw scores and times.

10.2196/71911 Checklist 1 CONSORT checklist.

## Abbreviations

CERAD: Consortium to Establish a Registry for Alzheimer Disease
CFS: Clinical Frailty Scale
CONSORT: Consolidated Standards of Reporting Trials
dNCR: delayed neurocognitive recovery
GDS-15: Geriatric Depression Scale-15
MMSE: Mini-Mental State Examination
NU-DESC: Nursing Delirium Screening Scale
p-NCD: postoperative neurocognitive disorder
SUS: System Usability Scale
SwQoR-24: Swedish Quality of Recovery questionnaire
WHODAS: World Health Organization Disability Assessment Schedule

## References

[1] Wolfe JD, Wolfe NK, Rich MW (2020). Perioperative care of the geriatric patient for noncardiac surgery. Clin Cardiol.

[2] Evered L, Silbert B, Knopman DS (2018). Recommendations for the nomenclature of cognitive change associated with anaesthesia and surgery-2018. Anesthesiology.

[3] Mahanna-Gabrielli E, Schenning KJ, Eriksson LI (2019). State of the clinical science of perioperative brain health: report from the American Society of Anesthesiologists Brain Health Initiative Summit 2018. Br J Anaesth.

[4] Amirpour A, Bergman L, Markovic G, Liander K, Nilsson U, Eckerblad J (2025). Understanding neurocognitive recovery in older adults after total hip arthroplasty-neurocognitive assessment, blood biomarkers and patient experiences: a mixed-methods study. BMJ Open.

[5] Borchers F, Knaak C, Piper SK, Spies C (2019). Recommendations for the detection and specification of perioperative neurocognitive disorders. Anasthesiol Intensivmed Notfallmed Schmerzther.

[6] Borchers F, Spies CD, Feinkohl I (2021). Methodology of measuring postoperative cognitive dysfunction: a systematic review. Br J Anaesth.

[7] Amirpour A, Eckerblad J, Bergman L, Nilsson U (2024). Comparing analog and digital neurocognitive tests with older adults: a study of the ISPOCD battery vs. a digital test battery from Mindmore. BMC Geriatr.

[8] van den Hurk W, Bergman I, Machado A, Bjermo J, Gustavsson A (2022). Swedish normative data for mindmore: a comprehensive cognitive screening battery, both digital and self-administrated. J Int Neuropsychol Soc.

[9] Hackett K, Xu S, McKniff M, Paglia L, Barnett I, Giovannetti T (2024). Mobility-based smartphone digital phenotypes for unobtrusively capturing everyday cognition, mood, and community life-space in older adults: feasibility, acceptability, and preliminary validity study. JMIR Hum Factors.

[10] Karlsson P, Nygren-Bonnier M, Henningsohn L, Rydwik E, Hagströmer M (2023). The feasibility of using a digital tool to enhance mobilisation following abdominal cancer surgery-a non-randomised controlled trial. Pilot Feasibility Stud.

[11] Lee KW, Hong YJ, Yang EJ (2024). Feasibility and usefulness of cognitive monitoring using a new home-based cognitive test in mild cognitive impairment: a prospective single arm study. BMC Geriatr.

[12] Skivington K, Matthews L, Simpson SA (2021). A new framework for developing and evaluating complex interventions: update of Medical Research Council guidance. BMJ.

[13] Amirpour A, Eckerblad J, Thorell A, Bergman L, Nilsson U (2023). Usability and feasibility of a digital cognitive screening tool measuring older adults’ early postoperative neurocognitive recovery: a protocol for a pilot study. BMJ Open.

[14] Lancaster GA, Thabane L (2019). Guidelines for reporting non-randomised pilot and feasibility studies. Pilot Feasibility Stud.

[15] Eldridge SM, Chan CL, Campbell MJ (2016). CONSORT 2010 statement: extension to randomised pilot and feasibility trials. BMJ.

[16] Lancaster GA, Dodd S, Williamson PR (2004). Design and analysis of pilot studies: recommendations for good practice. J Eval Clin Pract.

[17] Rockwood K, Song X, MacKnight C (2005). A global clinical measure of fitness and frailty in elderly people. CMAJ.

[18] Hörlin E, Munir Ehrlington S, Henricson J, John RT, Wilhelms D (2022). Inter-rater reliability of the Clinical Frailty Scale by staff members in a Swedish emergency department setting. Acad Emerg Med.

[19] Gaudreau JD, Gagnon P, Harel F, Tremblay A, Roy MA (2005). Fast, systematic, and continuous delirium assessment in hospitalized patients: the nursing delirium screening scale. J Pain Symptom Manage.

[20] Lingehall HC, Smulter N, Engström KG, Gustafson Y, Olofsson B (2013). Validation of the Swedish version of the Nursing Delirium Screening Scale used in patients 70 years and older undergoing cardiac surgery. J Clin Nurs.

[21] Myles PS, Weitkamp B, Jones K, Melick J, Hensen S (2000). Validity and reliability of a postoperative quality of recovery score: the QoR-40. Br J Anaesth.

[22] Nilsson U, Dahlberg K, Jaensson M (2017). The Swedish web version of the quality of recovery ccale adapted for use in a mobile app: prospective psychometric evaluation study. JMIR Mhealth Uhealth.

[23] Sheikh JI, Yesavage JA (1986). Geriatric Depression Scale (GDS): recent evidence and development of a shorter version. Clin Gerontol.

[24] Snellman S, Hörnsten C, Olofsson B, Gustafson Y, Lövheim H, Niklasson J (2024). Validity and test-retest reliability of the Swedish version of the Geriatric Depression Scale among very old adults. BMC Geriatr.

[25] Brooke J (1996). Usability Evaluation in Industry.

[26] Holmberg C, Gremyr A, Torgerson J, Mehlig K (2021). Clinical validity of the 12-item WHODAS-2.0 in a naturalistic sample of outpatients with psychotic disorders. BMC Psychiatry.

[27] Sekhon M, Cartwright M, Francis JJ (2022). Development of a theory-informed questionnaire to assess the acceptability of healthcare interventions. BMC Health Serv Res.

[28] Mbuagbaw L, Kosa SD, Lawson DO (2019). The reporting of progression criteria in protocols of pilot trials designed to assess the feasibility of main trials is insufficient: a meta-epidemiological study. Pilot Feasibility Stud.

[29] Nilsson U, Liander K, Rooyackers O, Eriksson LI (2019). Patients’ experiences of early postoperative cognition and its relation to cognitive decline and inflammatory responses: a protocol for a mixed-methods study. BMJ Open.

[30] Northgraves MJ, Arunachalam L, Madden LA (2020). Feasibility of a novel exercise prehabilitation programme in patients scheduled for elective colorectal surgery: a feasibility randomised controlled trial. Support Care Cancer.

[31] Macleod M, Steele RJC, O’Carroll RE (2018). Feasibility study to assess the delivery of a lifestyle intervention (TreatWELL) for patients with colorectal cancer undergoing potentially curative treatment. BMJ Open.

[32] Kyngäs H, Kaakinen P, Kyngäs H, Mikkonen K, Kääriäinen M (2020). The Application of Content Analysis in Nursing Science Research.

[33] Lewis JR (2018). The System Usability Scale: past, present, and future. Int J Hum Comput Interact.

[34] Murphy K, Kehoe B, Denieffe S, Hacking D, Fairman CM, Harrison M (2025). Comparing aerobic and resistance exercise emphasis during androgen deprivation and radiation therapy for prostate cancer: a randomised feasibility trial. Support Care Cancer.

[35] Boulanger MC, Lo SB, Centracchio JA (2025). Pilot feasibility trial of a supportive care digital application for patients with advanced nonsmall cell lung cancer. J Palliat Med.

[36] Ninomiya MM, Hiemstra J, Nicholson E, Isaac KV (2023). Methods of recruitment for surgical and perioperative randomized controlled trials: a rapid review. World J Surg.

[37] Katz MJ, Wang C, Nester CO (2021). T-MoCA: a valid phone screen for cognitive impairment in diverse community samples. Alzheimers Dement (Amst).

[38] Benge JF, Kiselica AM (2021). Rapid communication: preliminary validation of a telephone adapted Montreal Cognitive Assessment for the identification of mild cognitive impairment in Parkinson’s disease. Clin Neuropsychol.

[39] Brøgger-Mikkelsen M, Ali Z, Zibert JR, Andersen AD, Thomsen SF (2020). Online patient recruitment in clinical trials: systematic review and meta-analysis. J Med Internet Res.

[40] Lond B, Dodd C, Davey Z (2024). A systematic review of the barriers and facilitators impacting patient enrolment in clinical trials for lung cancer. Eur J Oncol Nurs.

[41] O’Brien KS, Harkins K, Peifer M (2025). Designing an intervention to improve cognitive evaluations in primary care. Implement Sci Commun.

[42] Perry M, Drašković I, Lucassen P, Vernooij-Dassen M, van Achterberg T, Rikkert MO (2011). Effects of educational interventions on primary dementia care: a systematic review. Int J Geriatr Psychiatry.

[43] Cartz-Piver L, Calvet B, Mehrabian-Spassova S (2023). Empowering general practitioners in dementia care: the ANTISTIGMA education intervention in Europe. Int J Geriatr Psychiatry.

[44] Chan LK, Chuan A, Berney CR, Chan DK (2024). Routine cognitive screening for older people undergoing major elective surgery: benefits, risks and costs. Anaesth Intensive Care.

[45] Hshieh TT, Yang T, Gartaganis SL, Yue J, Inouye SK (2018). Hospital elder life program: systematic review and meta-analysis of effectiveness. Am J Geriatr Psychiatry.

[46] Mackin RS, Burns E, Insel P (2025). Test taking location and practice effects as factors contributing to scores on remotely administered neurocognitive performance tests in a sample of older adults. Appl Neuropsychol Adult.

[47] Megari K, Kosmidis MH (2024). Protecting the brain while healing hearts: the protective role of cognitive reserve in cardiac surgery. Am J Geriatr Psychiatry.

[48] Feinkohl I, Winterer G, Spies CD, Pischon T (2017). Cognitive reserve and the risk of postoperative cognitive dysfunction. Dtsch Arztebl Int.

