# Supplementary material for: Evaluation of a Digital, Self-Administered, Cognitive Test Battery in Older Adult Patients Undergoing Abdominal Surgery: Nonrandomized Feasibility Trial
Source: JMIR Form Res. 2025 Nov 7;9:e71911. doi: 10.2196/71911 (PMC12594502; doi:10.2196/71911)
Supplement: Multimedia Appendix 1 [file formative-v9-e71911-s001.docx]

**Additional file 3. Interview guides**

**Interview guide: Study patients**

- How did you find using the tablet (Mindmore) before and after the surgery?
- How was your experience of undergoing cognitive assessment? Before and after surgery?

**Interview guide: Registered nurses involved in the feasibility study**

- Could you describe your role/function in this study?
- How do you perceive the recruitment of participants for this study? Is there anything that has worked well/less well? Why/why not?
- Compared to other clinical studies you have been involved in at your hospital, what barriers and opportunities have existed for recruitment in this study?
- Can you give examples of different strategies you have used to overcome any barriers or problems in recruiting study participants?
- Overall, do you perceive the study design (e.g., recruitment process, inclusion/exclusion criteria, and timing of inclusion/follow-up) to be feasible? Is there anything that has worked well/less well? Why/why not?
- Is there anything that has made you hesitant to ask patients about participating in this study? If so, what?
- How do you perceive communicating with our research group?
- How do you perceive communicating with R&D at the hospital?
- Can you describe what it has been like for you to participate in this study and measure and follow up the patient's cognitive function before and after surgery?
- What is your general impression of the digital cognitive testing tool Mindmore?
- What do you think is the value of measuring cognitive functions for patients undergoing surgery?
- What is your experience of assisting study participants in completing the test on their own, without the help of a test leader using Mindmore?
- What is your experience of using the Mindmore tablet as a tool for measuring cognitive functions?
- What do you think about the possibility of using the Mindmore tablet routinely in the clinic?
- Is there anything else you have thought about or have comments on about our study or Mindmore that you would like to share?
