# Supplementary material for: Evaluation of a Digital, Self-Administered, Cognitive Test Battery in Older Adult Patients Undergoing Abdominal Surgery: Nonrandomized Feasibility Trial
Source: JMIR Form Res. 2025 Nov 7;9:e71911. doi: 10.2196/71911 (PMC12594502; doi:10.2196/71911)
Supplement: Multimedia Appendix 2 [file formative-v9-e71911-s002.docx]

**Multimedia appendix.** Patients’ z-scores: data is compared with normative data based on birth year and sex.

| **Cognitive domain** | **T0, z-score, mean (SD)**  *n=24* | **95% CI** | **T1, z-score, mean (SD)**  *n=18* | **95 % CI** | **T2, z-score, mean (SD)**  *n=21* | **95% CI** | **T3, z-score mean (SD)**  *n=17* | **95% CI** | *p-value ∞* |
| --- | --- | --- | --- | --- | --- | --- | --- | --- | --- |
| **Memory**:  CERAD* | -0.45 (1.08) | -1.01, 0.17 | 0.18 (1.0) | -0.31, (0.67) | 0.29 (1.17) | -0.35, 0.82 | 0.07 (1.21) | -0.53, 0.78 | 0.51 |
| **Attention and tempo**  TMT A, SDPT | -1.18 (0.9) | -0.59, 0.19 | -0.55 (0.93) | -1.01, (-0.08) | -0.10 (0.81) | -0.47, 0.36 | 0.009 (0.7) | -0.35, 0.37 | 0.03 |
| **Executive functions*:**  TMT B*, Stroop | -0.2 (0.7) | -0.74, -0.11 | -0.33 (0.83) | -0.74, (0.08) | -0.08 (0.59) | -0.42, 0.21 | 0.1 (0.7) | -0.26, 0.47 | 0.02 |

Abbreviations: T0=baseline, T1=1-3 days postoperatively, T2=3-5 weeks postoperatively, T3=6 months postoperatively, CERAD=Consortium to Establish a Registry for Alzheimer Disease, TMT A, B = Trail Making Test Part A, Part B, SDPT=Symbols Digit Processing Test

*One missing at baseline. ***∞*** Friedman test

Depression, functional and quality of recovery scores

| **Outcome measure** | **T0 assessment**  **Mean (SD)**  *n=24* | **95% CI** | **T2 assessment**  **Mean (SD)**  *n=21* | **95% CI** | **T3 assessment**  **Mean (SD)**  *n=17* | **95% CI** | *p-value∞* |
| --- | --- | --- | --- | --- | --- | --- | --- |
| GDS-15 | 1.7 (1.8) | 0.93, 2.49 | 2.1 (1.9) | 1.15, 3.32 | 1.8 (1.7) | 0.86, 2.67 | 0.835 |
| SwQoR-24 | 37.65 (33.5) | 21.97, 49.65 | 34.8 (28.4) | 21.96, 45.66 | 33.7 (26.3) | 28.66, 55.34 | 0.359 |
| WHODAS 12 2.0 | 16.5 (4.6) | 14.09, 18.85 | 20.65 (8.2) | 16.43, 24.86 | 18.9 (6.3) | 15.64, 22.12 | 0.161 |

Abbreviations: GDS=Geriatric Depression Scale, QoR=Quality of Recovery Scale, WHODAS= World Health Organization Disability Assessment Schedule. ***∞*** Friedman test.
