# Supplementary material for: Evaluation of a Digital, Self-Administered, Cognitive Test Battery in Older Adult Patients Undergoing Abdominal Surgery: Nonrandomized Feasibility Trial
Source: JMIR Form Res. 2025 Nov 7;9:e71911. doi: 10.2196/71911 (PMC12594502; doi:10.2196/71911)
Supplement: Multimedia Appendix 3 [file formative-v9-e71911-s003.docx]

**Additional File 1. Cognitive raw scores for the different assessment points**

| **Name of cognitive test** | **Preoperative assessment**  **Mean (SD), median**  *n=24* | **Follow-up 1-3 days**  **Mean (SD), median**  *n=18* | **Follow-up 3-5 weeks**  **Mean (SD), median**  *n=21* | **Follow-up 6 months**  **Mean (SD), median**  *n=17* | ***p-value∞*** |
| --- | --- | --- | --- | --- | --- |
| CERAD learning | 17.42 (4.26)  Med:17 | 20.33 (3.10)  Med: 20.5 | 20.7 (4.75)  Med: 22 | 20.82 (4.23)  Med: 21 | 0.406 |
| CERAD delayed recall | 5.29 (1.96)  Med: 5 | 6.06 (2.57)  Med: 6 | 6.57 (2.65)  Med: 6 | 6.06 (2.58)  Med:6 | 0.18 |
| TMT A index | 0.55 (0.19)  Med: 0.57 | 0.54 (0.14)  Med: 0.51 | 0.58 (0.17)  Med: 0.54 | 0.64 (0.17)  Med: 0.61 | 0.116 |
| Time (s) | 49.96 (23.59)  Med: 42 | 46.19 (12.81)  Med: 47 | 44.1 (12.3)  Med: 44 | 40.2 (10.85)  Med: 39 | 0.104 |
| Correct connections | 23.83 (0.48)  Med: 24 | 23.81 (0.54)  Med: 24 | 23.95 (0.21)  Med: 24 | 24 (0)  Med: 24 | 0.145 |
| TMT B index | 0.18 (0.08)  Med: 0.17 | 0.19 (0.23)  Med: 0.22 | 0.22 (0.09)  Med: 0.24 | 0.25 (0.08)  Med: 0.26 | 0.005 |
| Time (s) | 113.09 (33.25)  Med: 116 | 106.13 (50.28)  Med: 99 | 98.52 (41.48)  Med: 88 | 95.53 (41.77)  Med: 92 | 0.014 |
| Correct connections | 19.68 (5.76)  Med: 22.5 | 19.25 (6.63)  Med: 22 | 20.33 (6.21)  Med: 24 | 21.88 (4.6)  Med: 24 | 0.011 |
| Stroop incongruent index | 8.65 (2.1)  Med: 8.4 | 8.80 (2.56)  Med: 8.82 | 9.81 (2.08)  Med: 9.7 | 9.72 (2.48)  Med: 9.4 | 0.064 |
| Stroop interference (ms) | 743 (568)  Med: 614 | 442 (860)  Med: 325 | 592 (386)  Med: 480 | 462 (621)  Med: 518 | 0.109 |
| Stroop congruent average (ms) | 1625 (464)  Med: 1573 | 1914 (585)  Med: 1863 | 1475 (242)  Med: 1494 | 1673 (415)  Med: 1606 | <0.001 |
| Stroop incongruent average (ms) | 2368 (585)  Med: 2214 | 2356 (782)  Med: 2155 | 2080 (434)  Med: 2051 | 2135 (523)  Med: 2109 | 0.026 |
| SDPT correct | 31.17 (4.97)  Med: 31.5 | 27 (7.6)  Med: 28 | 32.14 (5.86)  Med: 32 | 31.65 (5.86)  Med: 33 | 0.005 |
| SDPT incorrect | 0.96 (1.26)  Med: 0.5 | 1.06 (1.2)  Med: 1 | 0.95 (1.32)  Med: 1 | 1.18 (1.59)  Med: 1 | 0.405 |

***∞***Friedman test
